# Coming on Its Own, Global Oncology Research

**DOI:** 10.1200/JGO.18.00025

**Published:** 2018-03-15

**Authors:** Ophira Ginsburg, Gilberto Lopes

**Affiliations:** **Ophira Ginsburg**, New York University Langone Health, New York, NY; and **Gilberto Lopes**, Sylvester Comprehensive Cancer Center, Miami, FL

What is global oncology? In the inaugural editorial for *Journal of Global Oncology* (JGO), past ASCO Presidents, Peter Yu and Julie Vose, cited Sandra Swain’s call to build bridges in cancer globally, and proposed that JGO would serve to build connections to reduce cancer health disparities between wealthier and poorer countries, and within high-income countries.^1^ Moreover, they suggested that this new journal would not only provide a vehicle for publishing high-quality research from low- and middle-income countries, but it would also focus on any subject matter that would better “reflect the unique biologic and social attributes of these populations.”^1^ It would do so “by enabling ASCO members to contribute to the vitally important and dynamic global conversation on health care, including access to care, quality of care, and perhaps most important, the design of systems of health care delivery that affect populations of patients with cancer”.^1^ As JGO founding editor David Kerr noted, important discoveries and approaches to cancer control have been achieved by oncologists and scientists from low- and middle-income countries not despite, but precisely because of, limited resources. Necessity continues to be the mother of invention.^2^

The first issues of JGO helped define the field of global oncology—a new framing of our global discourse on cancer research. Global oncology is inclusive not only of people and places, but of the widest range of topics and methodologies, which reach beyond the traditional scope of most academic cancer journals. Global oncology research must therefore include investigations of the upstream and downstream factors that can influence individual and population differences in cancer risk, incidence, mortality, and survival, as well as quality of life. In many ways, global oncology is at the forefront of this re-imagining of the cancer control continuum, encompassing domains that are addressed in other areas of global health, such as the social, economic, and the so-called commercial determinants of health.^3^ As the costs of new cancer medicines continue to skyrocket, and in the era of precision medicine in which most newly approved oncology drugs are of relatively limited clinical benefit, all countries, regardless of gross domestic product, must ask the question: What can we afford, and what do we mean by value?^4^

That an equity lens is at the core of JGO is not surprising. ASCO is the largest global association for clinical oncology, with 44,000 members working in more than 150 countries. Last year’s ASCO Annual Meeting welcomed more than 30,000 cancer professionals, nearly 50% of whom came from outside the United States.^5^ In 1997, the ASCO International Affairs Committee was formed, noting the potential for a truly global role in cancer care and control. A recent issue of ASCO Connection described the growing role of the organization in global oncology training, research, and mentorship.^5^ To highlight a few of these initiatives, the past 15 years saw the initiation of the International Development and Education Awards, the Conquer Cancer Foundation International Innovation Grants program, and the new Virtual Mentor Program.^6^ In 2014, the ASCO Board of Directors appointed a Global Oncology Leadership Task Force to provide recommendations for further expansion ASCO’s international programs and contributions in global oncology.^7^ In 2018, Conquer Cancer added the Global Oncology Young Investigator Award to its portfolio of grants and awards.

Partnerships are at the core of ASCO’s global oncology efforts. Whether North/South, South/South, or a more complex arrangement between organizations, institutions, and academies, partnerships are fast becoming the main route to success not only in research collaborations, but also in cancer control efforts more broadly. There is much we can learn from the broader global health community. Historically focused on public health and factors that influence mortality from infectious diseases, maternal/child health, and its related topics in health systems and health care delivery, the modern view of global health is comprised of health economics, financing, climate, and ecology, leading to the paradigm of planetary health.^8^

Global health—the health of populations in the global context^9^—is “the area of study, research, and practice that places a priority on improving health and achieving equity in health for all people worldwide”^10^^(p1993)^. Global health is about worldwide health improvement, the reduction of disparities, and protection against global threats that disregard national borders.^11^ Global health is not to be confused with international health, which is defined as the branch of public health that focuses on developing nations and foreign aid efforts by industrialized countries.^12^

Cancer researchers from countries at all income levels are driven by the same urgent need to reduce cancer health disparities and to innovate via translational research; service delivery; health systems solutions, such as by task shifting and task sharing; with novel financing or procurement models; and in efforts to repurpose drugs, to name only a few examples. Since its inaugural issue, JGO has published numerous high-quality reports in cancer causation, care, and control that have been inspired by the wider community of global health researchers.

The 6th Annual Symposium on Global Cancer Research (ASGCR) will take place on March 15, 2018, in New York, NY, and is expected to draw a diverse group of participants from five continents. This year’s symposium is being organized and sponsored by the National Cancer Institute Center for Global Health, Perlmutter Cancer Center and NYU Langone Health, the Consortium of Universities in Global Health (CUGH), with cosponsors that include the Albert Einstein College of Medicine, Herbert Irving Comprehensive Cancer Center at Columbia University, Memorial Sloan Kettering Cancer Center, the Tisch Cancer Institute, Icahn School of Medicine at Mount Sinai, Rutgers Global Health Institute, and the Rutgers Cancer Institute of New Jersey. The theme of this year’s symposium—Global Cancer Research: Addressing Disparities, Locally and Globally*—*is aligned with the CUGH 9th Annual Meeting, of which ASGCR is a satellite event. An editorial by Kostelecky et al^13^ that accompanies this commentary describes the history of the relationship between the National Cancer Institute and CUGH, as well as its growing collaboration with ASCO. As in previous years, JGO will publish selected abstracts presented at this year’s symposium.

Since its first year, JGO has received 857 submissions and published 197 articles, all of which are now listed on PubMed. The opportunities for individuals and organizations to engage in this global conversation to control cancer and reduce cancer health disparities have never been greater. Although this broader discourse on cancer control has been slow to evolve, we are perhaps entering a golden age. As such, we encourage everyone to read this year’s selected abstracts from the 6th ASGCR.

*“We can’t think of ASCO’s role in global oncology as a form of foreign aid,” said Dr. Hortobagyi. “We should think of these efforts as an approach to continuously improve the quality and quantity of both cancer research and cancer care in different parts of the world, including the United States.”*^5^
